# Q Fever in Young Children, Ghana

**DOI:** 10.3201/eid1402.070971

**Published:** 2008-02

**Authors:** Robin Kobbe, Stefanie Kramme, Benno Kreuels, Samuel Adjei, Christina Kreuzberg, Marcus Panning, Ohene Adjei, Bernhard Fleischer, Jürgen May

**Affiliations:** * Bernhard Nocht Institute for Tropical Medicine, Hamburg, Germany; † Kumasi Centre for Collaborative Research in Tropical Medicine, Kumasi, Ghana; 1 These authors contributed equally to this study.

**Keywords:** Q fever, Coxiella burnetii, malaria, seroprevalence, antibody, children, fever, IFA, Ghana, letter

**To the Editor:** Recently, experts identified Q fever, caused by the small, gram-negative bacterium *Coxiella burnetii*, as an important underdiagnosed childhood disease (*1*). Studies on Q fever in children <5 years of age are scarce, especially with respect to sub-Saharan Africa. The only available study from Niger reports a seroprevalence of 9.6% (*2*). Throughout Africa, prevalence of Q fever in adults shows considerable variability and is highest in countries with prominent stockbreeding (*3*).

Clinical manifestations of Q fever in children are similar to those of malaria (*1*,*4*). In malaria-endemic areas, most fevers are attributed to *Plasmodium falciparum* infection and presumptively treated with expensive combination therapies (*5*). In this context, other neglected fever-causing pathogens need to be given appropriate consideration.

We studied the prevalence of Q fever antibodies in 219 randomly selected children living in 9 rural villages of the Ashanti region, Ghana. Plasma was obtained by venous puncture from 2-year-old children after they had participated in a malaria control study and had been clinically monitored for 21 months. Clinical, parasitologic, socioeconomic, and Global Positioning System information was recorded as described elsewhere (*6*,*7*). In addition, 158 healthy adult volunteers from the same area were included. Plasma was stored at –20°C until microimmunofluorescence assays (IFA) (*Coxiella burnetii* I+II, Vircell SL Microbiologists, Granada, Spain) were performed according to manufacturer’s instructions. To identify all children with Q fever titers, we regarded the following as positive fluorescence reactions to plasma dilutions: >1:64 for phase II immunoglobulin (Ig) G and >1:24 for phase II IgM with sensitivity (specificity) of 97.2% (100%) and 100% (56.3%), respectively. IgM testing was only performed on IgG-positive children. Positive and negative controls were run on each IFA slide. Relative risks (RR) for characteristics of children were calculated by χ^2^ test; p<0.05 was considered significant. Informed consent was obtained from all participants or their parents. The study protocol was approved by the committee on human research and publication, Kwame Nkrumah University of Science and Technology, Kumasi, Ghana.

Positive *C. burnetii* phase II IgG responses were observed in 37 (16.9%) of 219 children and 14 (8.9%) of 158 adults (Figure, panels A and B). In comparison to adults, more children had IgG titers >64 (Figure, panels C and D). On the day of the serosurvey 71 (32.4%) of 219 children had fever (measured body temperature >38°C or reported fever within the previous 48 hours). Test outcome did not appear to be influenced by *P. falciparum* infection, since 4 of 37 IgG-positive children (23 of 182 IgG-negative children) had clinical malaria, 11/37 (62/182) had asymptomatic parasitemia, and 6/37 (38/182) had fever without parasitemia, and there were no significant differences between groups. The frequency of prior malaria episodes also did not influence antibody response. Three aparasitemic children had positive phase II IgM titers (24, 96, and 1,536; phase II IgG 64, 64, and 4,096, respectively). The child with the high IgM and IgG titers was clinically ill with nonsevere *C. burnetii* pneumonia. This child was among 10 (27%) of 37 phase II IgG-positive children with detectable anti–*C. burnetii* phase I antibodies. Of all sociodemographic characteristics under consideration, only maternal illiteracy was associated with positive phase II IgG testing (RR 2.1, 95% confidence interval 1.0–4.2, p<0.05).

A considerable proportion of Ghanaian children had anti–*C. burnetii* antibodies, which indicates that Q fever might be a common event in this age group. Antibodies were more frequently detected in children than in adults. In adults, Q fever IgG antibodies reach a maximum 4–8 weeks after onset of symptoms and gradually decrease over months to finally fall below the detection limit (*8*).

A long period since infection is less likely in young children, which could result in higher seropositivity. Children, especially those of illiterate mothers, could also be more frequently exposed to the pathogen. Consumption of unpasteurized dairy products can result in infection or seroconversion without clinical disease (*9*). However, because consumption of raw milk in the Ashanti region is regarded as being uncommon by local health authorities, we consider dairy products an unlikely source of the disease. Although participants were intensively exposed to *P. falciparum,* which causes polyclonal B-cell stimulation, malaria episodes and parasitemia with and without symptoms at time of the serosurvey did not influence testing (*10*). This finding is important because commercially available test kits have only been evaluated in Europeans not exposed to parasites. We cannot completely rule out the possibility that other infectious agents, which are either only prevalent or more prevalent in African populations, could have resulted in false-positive results. Nevertheless, the test method we used and existing data on cross-reactions weaken this hypothesis (*8*).

We conclude that children in rural sub-Saharan Africa become exposed to *C. burnetii* early in life and that Q fever, which is clinically indistinguishable from malaria, may develop in an unknown proportion of them. The incidence of Q fever in relation to malaria, the route of infection, and appropriate serologic cutoffs for sub-Saharan Africa must be defined further. Currently, a prospective diagnostic study is investigating neglected infections, including human Q fever, as a cause of illness in Ghanaian children.

**Figure:**
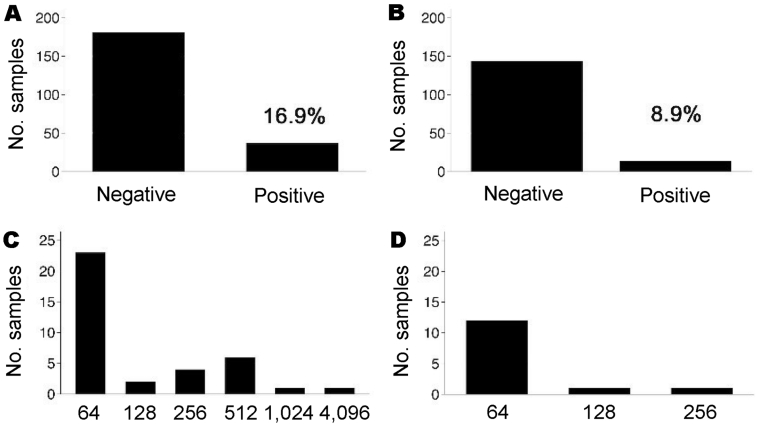
Seroprevalence of immunoglobulin (Ig) G antibodies against *Coxiella burnetii* phase II tested by microimmunofluorescence assays (IFA). A) Results of serologic tests of children, a cutoff titer of >64 for *C. burnetii* phase II IgG was applied; B) results of serologic tests of healthy adults (cutoff >64); C) distribution of *C. burnetii* phase II IgG titers in all positive children; D) distribution of IgG titers in all positive healthy adults.
